# Conspecific injury raises an alarm in medaka

**DOI:** 10.1038/srep36615

**Published:** 2016-11-08

**Authors:** Ajay S. Mathuru

**Affiliations:** 1 Yale-NUS College, 12 College Avenue West, #01 - 201, – 138610, Singapore; 2 Mechanisms Underlying Behavior, IMCB, 61 Biopolis Way, - 138673, Singapore

## Abstract

In the late 1930s, Karl von Frisch reported that semiochemicals released upon injury, act as alarm substances (*Schreckstoff*) in fish. In Ostariophysi species, club cells in the epidermis are believed to contain cues related to alarm substance; however, the function of club cells, primarily as reservoirs of alarm substance has been debated. Here, I describe an alarm response in the Japanese rice fish *Oryzias latipes* (medaka), a member of the order Beloniformes. The response to alarm substance (*Schreckreaction*) in medaka is characterized by bouts of immobility and an increase in cortisol levels within minutes of exposure to conspecific skin extract. Histological analysis, however, suggests that club cells are either rare or absent in the medaka epidermis. In addition to describing an uncharacterized behavior in a vertebrate popular for genetic and developmental studies, these results support the hypothesis that the primary function of epidermal club cells may be unrelated to a role as alarm substance cells. The existence of similar behavioral responses in two evolutionarily distant but well established laboratory models, the zebrafish and the medaka, offers the possibility of comparative analyses of neural circuits encoding innate fear.

Avoiding predation is critical for survival. Many plants and animals communicate danger of predation via a multitude of methods, including alarm calls, signals and chemical cues^1^,^2^,^3^. Fish are also known to be alerted to danger by different chemical cues that include predator odors and disturbance cues from conspecifics (see^4^,^5^,^6^,^7^ for some recent examples). The first report of a chemical alarm signal in fish dates back to 1938^8^. Karl von Frisch observed that a substance, or *Schreckstoff* (panic substance in German) released after a physical injury to a minnow (*Phoxinus phoxinus*) elicited a stereotypic *Schreckreaktion (*panic or fright reaction in German) in other, nearby minnows. Subsequently, other researchers have described conspecific injury induced alarm in other aquatic taxa, including fish from other families, as well as marine snails, tadpoles and sea urchins^9^,^10^,^11^. The list of aquatic animals that show such responses has significantly expanded since Karl von Frisch’s discovery (see^4^,^12^ for a comprehensive list). Among these, the behavior and the neural mechanisms that mediate the response of fish in the superorder Ostariophysi, where this phenomenon was first described, has been covered extensively in the literature^13^. The observation that fish have evolved to interpret bodily harm to a conspecific as a sign of danger, has fascinated researchers for decades. As this form of communication is not entirely species specific, is involuntary, and often occurs after a fatal injury to the originator, the evolutionary pressures that maintain alarm substance production and its storage have been a subject of debate (see^14^,^15^,^16^,^17^,^18^).

For instance, it has been difficult to find empirical evidence of increased fitness of the sender for apparently maintaining a set of “alarm substance cells” to synthesize and store alarm substance specifically to warn other conspecifics^17^,^18^,^19^,^20^. A population of large secretory cells with fine fibrils in their cytoplasm, called the club cells^21^, are thought to perform this function in Ostariophysans^22^,^23^. Club cells are located in the intermediate or superficial layers of the epidermis of most Ostariophysan species and present with a characteristic central nucleus that distinguishes them from other large cells, like the mucous or the granule cell. Club cells are expected to be energetically expensive to maintain^24^, and, as such, their sole function as an early warning system for others raises questions of adaptive benefit for the sender.

How did the club cells come to be associated with the alarm response in the first instance? The oldest reference goes back to W. Pfeiffer. In the early 1960s, W. Pfeiffer, a student of von Frisch, suggested that a type of large secretory club cell (*Kolbenzellen*) in the teleost skin that do not open on the epidermal surface, are associated with alarm substance and termed them *Schreckstoffzellen* (or fright substance cells; reviewed^22^). He arrived at this conclusion having collated observations of fright response, or the lack of it, in species of fish from over 70 families. The fright reaction appeared to be independent of habitat, but mainly restricted to species in the orders Ostariophysi and Gonorynchiformes among the teleost^23^. He noted that a conspicuous difference between the skin of species that produced an alarm substance and those that he considered did not, was the presence of a distinctive secretory cell type (club cells) close to the outer surface of the skin^23^. In addition, he also noted that non-Ostariophysi fish, particularly marine fish or those that had fewer, smaller, or a complete absence club cells, did not display a fright reaction^23^,^25^. The presence of club cells was considered morphologically unique at that time and became one criteria for merging Ostariophysi and Gonorynchiformes into a single superorder^23^.

This interpretation and the use of term *Schreckstoffzellen* to describe club cells was not accepted by all^26^. Several other caveats are also visible in this interpretation now, that suggest the need to revisit this association. For example, whilst club cells are often used synonymously for presence of alarm substance and vice versa^27^,^28^ other types of cells have also been implicated as potential sources for damage-released alarm cues^5^. In addition, the alarm response to conspecifc injury has been described in non-ostariophysan species that may not possess club cells^4^,^12^,^29^,^30^,^31^. Further, oligomers of a chondroitin-like molecule have been identified as components of the alarm substance mixture that elicit alarm in zebrafish^32^. Chondroitin-like molecules, though present in club cells^33^, are not limited to these cells in fishes. Finally, at least two sets of independent experiments suggest that club cells are an integral part of an innate immune system in fishes^17^,^34^. Epidermal club cell density is increased by skin-penetrating parasites and pathogens, but appears to be uncorrelated with a change in predation risk of a species^17^. However, extract from the skin of species that have club cells, inhibits the growth of a parasitic protozoan, *Saprolegnia*^17^. These authors have thus proposed that Ostariophysan club cells may have been selected over evolutionary timescales for their immune function in defence against pathogens, parasites, or UV irradiation induced damage, and their role in alarm response could be peripheral or acquired secondarily.

One possible outcome of this alternate hypothesis is the likelihood of the existence of fishes that display a fright reaction but have few, or no club cells. Club cells were primarily described in species from the superorder Ostariophysi. I explored if *Oryzias latipes* (Medaka) from the series Atherinomorphae, display an alarm response and if they have club cells in their skin. Medaka have been used both as pets and as laboratory animals for over a century. An incredibly rich scientific literature (largely in Japanese) dating back to the late 18th and early 19th century, suggests early adaptation of this rice field fish for studying genetic transmission of traits (for a review see^35^ and^36^). Surprisingly however, records of studies of alarm or other behaviors are not easy to find.

Here, I show that medaka display a distinctive alarm response after exposure to conspecific skin extract. Apart from the external behavioral changes, this exposure is physiologically arousing as it activates the stress response pathway resulting in an elevation in the whole-body cortisol levels. Though conspecific skin extract elicits a fright reaction, the skin of medaka lacks a presence of discernible club cells. These results together suggest that epidermal club cells are not necessary for production of alarm substance in medaka.

## Results

### Medaka display an alarm response

I used video recordings and automated tracking to quantify if the swimming behavior of adult medaka changes upon exposure to conspecific skin extract (henceforth referred to as CSE; please see methods for details). In a pilot experiment using 2 adults each as experimental (exposed to CSE; Video S1) and control (exposed to fish water) subjects, I found that after exposure to CSE, at least two of the three parameters previously described as characteristics of alarm behavior of fish reared in laboratories^32^,^37^, were evident in experimental subjects (Supplementary Figure S1). Swimming patterns of the subjects did not change during, or immediately after, the delivery of the CSE, but became evident after a delay of two to three minutes. There was no increase in erratic swimming (darting) or a descent to the bottom of the tank as described in CSE exposed minnows and zebrafish^8^,^38^; however, there was an abrupt change, wherein fish stopped swimming actively. Similar alarm behavior has been described in others^30^. Medaka appeared to drift passively, sometimes towards the bottom of the observation tank (Video S1). The episodes of immobility with intermittent resumption of normal swimming, lasted for the duration of the assay (remaining eight minutes).

**Figure 1:**
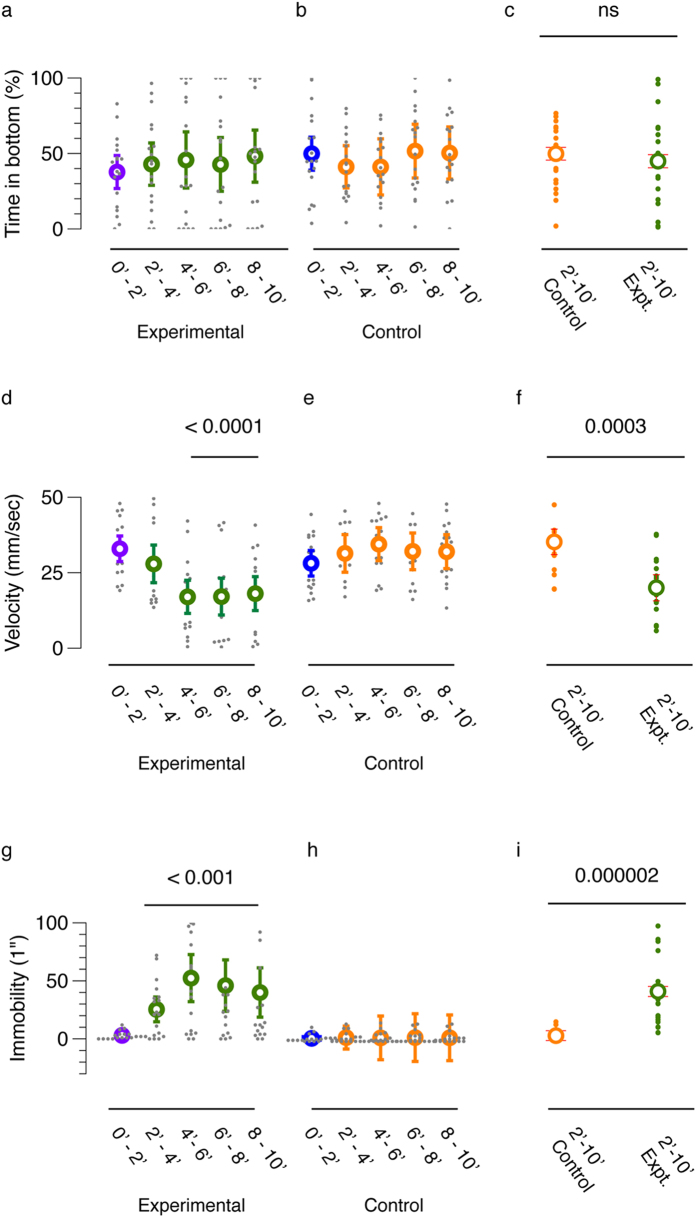
Behavioral parameters of alarm response to conspecific skin extract in medaka. Behavioral measures before (purple; blue) and after (green; orange) for experimental (exposed to CSE) and control subjects respectively. n = 18. (**a–c**) show percentage of time spent in the bottom quarter, (**d–f**) show velocity in mm/sec, (**e–g**) show number of immobility episodes. Behavior measures in 2-minute observation time bins in (**a,d,g**) are for experimental and (**b,e,h**) are for control subjects. (**c,f,i**) show comparison of control and experimental subjects for the same parameters. Error bars show 95% confidence intervals. Mean is shown in the foreground and population disctribution is shown in the background. P values in (**d**,**g**) are from Kruskal-Wallis H test and in (**c**,**f**) and **i**) are from Mann-Whitney U test.

To quantify these parameters, I repeated the experiment with 18 control and 18 experimental subjects. As the response in the pilot experiment did not appear immediately, I quantified behavioral parameters in two minute time-bins. Stimulus was delivered after two minutes (experimental: green, control: orange, Fig. 1). The mean position of experimental or control subjects did not change substantially after delivery of the stimulus over the duration of the assay (Fig. 1a,b). There was no significant difference between control and experimental subjects in the post-stimulus period (Fig. 1c).

The average swimming velocity, decreased in experimental subjects and not in control subjects (Fig. 1c,d). A Kruskal-Wallis H test revealed a statistical difference between the observation time-bins in the experimental condition (Fig. 1d; KW H statistic = 22.23, p < 0.0001). Pairwise comparisons using a nonparametric Mann-Whitney U test, revealed that compared to the first (0′–2′: before) time-bin, velocity decreased in the third, fourth and fifth the time-bins (Fig. 1d; all Us < 54.0, all ps < 0.001). The post-stimulus comparison between control (mean = 32.4 mm/sec, 95%CI [29.0 mm/sec, 35.8 mm/sec]) and experimental subjects (mean = 20.0 mm/sec, 95%CI [15.7 mm/sec, 24.3 mm/sec]) for velocity, also revealed a statistically significant difference (M-W U = 49.0, p = 0.0003; effect size Cohen’s d = 1.49). This reduced velocity could be explained in part by an increase in the number of episodes of immobility in experimental subjects (see methods for description of an immobility episode; Fig. 1g). Again, a Kruskal-Wallis H test revealed a statistical difference between the observation time-bins in the experimental condition (Fig. 1g; KW H = 20.6, p < 0.001) but not in the control condition. Pairwise comparison revealed an increase in the episodes of immobility in all the time-bins compared to the first (0′–2′: before) time-bin (Fig. 1g, all M-W Us < 54.0, all ps < 0.001). The post-stimulus period comparison between control (mean = 3.2, 95% CI [1.3, 5.1]) and experimental subjects (mean = 40.8, 95%CI [35.5, 56.3]) for immobility also revealed a statistically significant difference (M-W U = 13.0, p = 0.000002; effect size Cohen’s d = 1.5).

These results suggest that medaka exposed to CSE (experimental condition) respond by swimming slowly and by becoming immobile for extended periods.

### Exposure to conspecific skin extract raises cortisol levels

**Figure 2:**
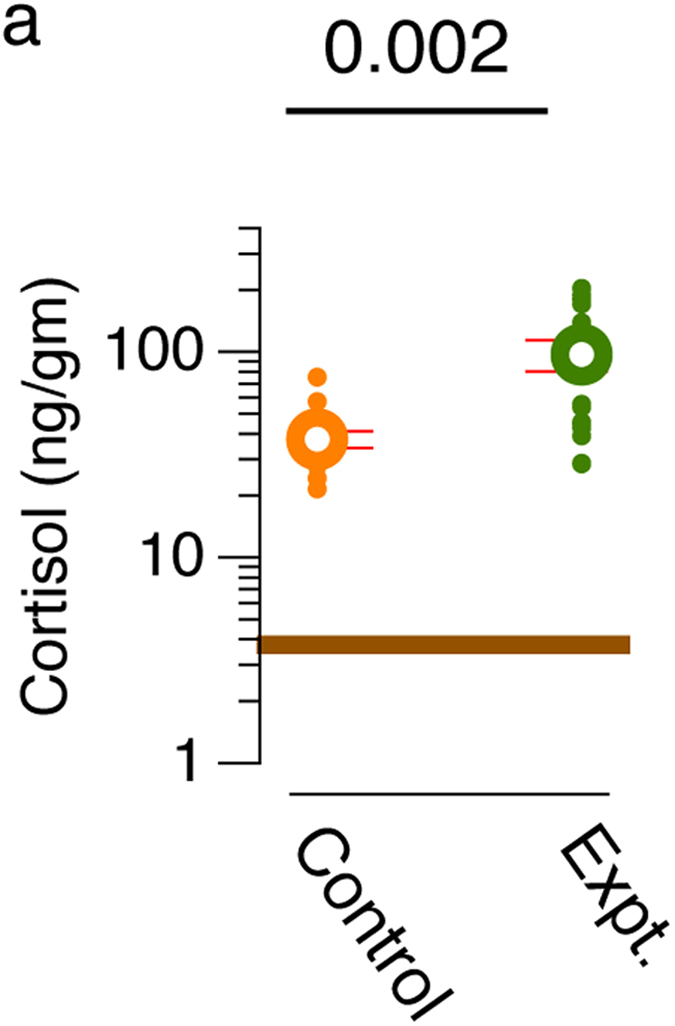
Cortisol measures. Whole-body cortisol (ng/gm of body weight represented on a log-scale) measured from controls subjects or those exposed to CSE (n = 16). Brown line indicates cortisol levels of fish from home tank (n = 8). Error bars show 95% confidence intervals. Mean is shown in the foreground and population disctribution is shown in the background. P value from Mann-Whitney U test.

As has been observed in other vertebrates, the circulating cortisol levels change in fish when they are emotionally aroused, anxious, or stressed. Cortisol levels change via activation of the hypothalamus-pituitary-interrenal axis and peak within 15 minutes^39^,^40^. Exposure to CSE can be a stressful event for some fish^41^,^42^,^43^. I asked if behavioral changes in experimental subjects, described above, is accompanied by changes in cortisol levels. To address this question, I measured whole-body cortisol levels from the experimental and control subjects (N = 16 fish/condition). Baseline cortisol (mean = 3.1 ng/gm, 95%CI [2.3 ng/gm, 3.9 ng/gm]) was obtained from fish taken directly from the home tank at the time of day when experiments were conducted (indicated by brown line in Fig. 2). Compared to control subjects (mean = 37.5 ng/gm, 95%CI [34.1 ng/gm, 41.1 ng/gm]; Fig. 2) experimental subjects exposed to CSE, showed an increase in whole-body cortisol levels (mean = 97.0 ng/gm, 95%CI [80.1 ng/gm, 113.9 ng/gm]; M-W U = 47.0, p = 0.002; effect size Cohen’s d = 1.2). As such, in addition to behavior indicative of fear, whole-body cortisol levels also increase in medaka exposed to CSE.

### Epidermal club cells are difficult to detect in medaka

The experiments above suggest that medaka have an alarm response to CSE. As described earlier, alarm substance containing club cells were considered abundant, but unique to Ostariophysi that show a fright response^23^. I asked whether a non-ostariophysan species that display an alarm response have club cells in their epidermis. Epidermal club cells do not have a positive marker that can be used to identify them; however, morphological characteristics have been used in the past to distinguish these cells from the other three main categories of secretory and large cells. These include - a) location in the middle or intermediate layers, b) no opening to the surface, c) a round, oval or club shape, d) a central nucleus though sometimes binucleate, and e) fibrillar cytoplasm that is negative for Periodic Acid Schiff (PAS) staining^23^,^26^,^28^,^44^.

**Figure 3:**
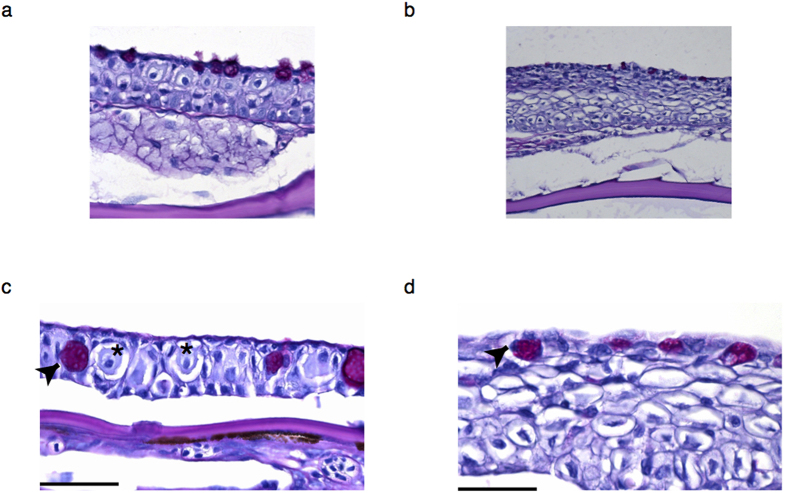
Histology of epidermis of medaka and zebrafish. Low (**a,b**) and high magnification (**c,d**) images of the zebrafish (**a,c**) and medaka (**b,d**) epidermis tissue. Club cells in the zebrafish epidermis (**c**) are negative for PAS staining and are indicated by an asterisk (*). Arrowheads show another type of secretory cell that is positive for PAS staining - the mucous goblet cell. Scale bar is 50 um.

In Ostariophysi, histological sections of fish skin prepared with PAS and hematoxylin counterstaining, show club cells interdigitated with other cell types in the intermediate layers^17^,^28^. Figure 3a,c show examples from zebrafish at two magnifications. These cells are approximately 20–25 um in diameter (asterix) with a prominent central nucleus. The same procedure however, failed to reveal morphologically matching cell type in any of the medaka examined (Fig. 3b,d, Supplementary Figure 2). Other secretory cells such as the goblet mucous cell (arrowhead) could be detected in both zebrafish and medaka, albeit there were differences in numbers and size.

## Discussion

In this set of experiments, I show that like many Ostariophysi, medaka also display an alarm response to chemical cues released upon injury to conspecifics. In medaka, the alarm response is characterized by episodes of immobility or freezing, interspersed with normal swimming that lasts for at least ten minutes. There is an increase in circulating cortisol, measureable around ten minutes after exposure to CSE; however, club cells are difficult to detect. This is not surprising, as conspicuous club cell presence has been considered a unique morphological feature of Ostariophysi. Since no positive markers for club cells are known, it is however not possible to conclude with certainty that they are absent in medaka. It remains possible, for instance, that they are present in fewer numbers, or are smaller, or have morphological differences compared to those observed in Ostariophysi.

Club cells are being recognized as a evolutionary innovations that likely serve several functions^17^. Apart from the well-known proposal for storing alarm substance^23^ other proposed roles include attracting secondary predators^18^,^19^, defense against pathogens/parasites^17^,^34^, wound healing^45^ and phagocytosis^46^. Given this diversity of plausible functions, some researchers have argued that their characterization as *Schreckstoffzellen* is a misnomer and should be avoided^26^.

In support of this argument, the results shown here add to a fairly long list of other non-Ostariophysan fish that show an alarm response to injury-released chemicals, including darters (family Percidae), gobies (family Gobiida), sticklebacks, sculpins (*Cottus cognatus*), and guppies (*Poecilia reticulata*)^12^,^15^,^31^,^47^. However, as seen in medaka here, club cells are either rare or absent in some of the species examined^5^,^48^,^49^. In aaddition, anti-predator responses have been described in fathead minnows (*Pimephales promelas*) to alarm substance prepared from conspecific larvae that lacked visible club cells^50^. Taken together, these suggest that detectable, or specialized epidermal cells are ancilliary to production of alarm substances in either ostariophysans or non-ostariophysans.

Medaka in the experimental condition here, show an increase in whole-body cortisol level that is higher than that observed due to handling and isolation from the shoal during experimentation (controls), suggesting that exposure to conspecific injury-released substances is stressful. The relatively low levels of cortisol in fish from the home tank compared to controls (Fig. 2; M-W U = 0, p < 0.001) indicates that the process of behavior recording is in itself stressful, and exposure to CSE is an additional stressor. Activation of the primary stress response pathway in reaction to CSE exposure, as seen in medaka, has also been reported in many other species^41^,^42^,^43^,^51^,^52^. There are examples, however, (*Brycon cephalus* and *Rhamdia quelen*) where conspecific injury elicits an anti-predator behaviour, but cortisol changes are undetectable^53^,^54^. It is possible that alternate stress response pathways are triggered in some species, or in specific contexts^6^,^43^. It is also conceivable that detection thresholds in cortisol changes are subject to technical limitations as stess due to experimental processes may mask the stress from CSE exposure. This suggests the need for further experiments in these and/or other species to evaluate context in which (or even the species where) antipredator behavior is related to activation of the HPI stress pathway.

These results open up the possibility of comparative analysis of neuroanatomy and neural circuit function in zebrafish and medaka, as both species are amenable to *in vivo,* whole brain neural activity studies using genetically encoded neural activity reporters (calcium and voltage indicators). Alarm response to conspecific injury could have originated multiple times, or once in a common ancestor in fishes. Comparative studies of behavior, biochemistry of alarm cues, and neural circuits of these two species that shared a common ancestor approximately 110–160 Myr ago^55^, will be highly informative. Though this is an exciting possibility, one should note that the experiments described in this study were performed on a single inbred strain of medaka (cab) commonly used in the laboratory. They will need replication in other laborator strains and in the wild.

Finally, as suggested earlier by many, including RJF Smith, D Chivers, R Mirza and B Wisenden among others, the evolution of injury-released substance as alarm cues may not be so unusual. The selection pressure likely operates on the receivers who respond to alarm substance. It is in the interest of the reciever to evolve mechanisms to reliably detect presence of predators when other prey fish are under attack. Conversely, special mechanisms are not required for the alarm cues or their storage apparatus (like the Ostariophysi club cells) to evolve in the sender for this purpose. Substances, or cells like the club cells that have other primary functions, could acquire a role in the alarm response secondarily in such a context.

## Methods

Experiments were performed in accordance to the guidelines recommended by the Institutional Animal Care and Use Committee (IACUC) of the Biological Resource Center at A*STAR. Approved experimental protocols (IACUC # 161110) were followed.

### Behavior

Medaka from the inbred line ‘cab’ were used for all experiments described. The line was a gift of Christoph Winkler. Embryos obtained were raised in-house. Subjects were tested individually, between 13:00 and 18:00 in the afternoon. Experiments were conducted on adults aged between 4–8 months. Most experimental subjects were males due to limited availability of female fish. For testing, a net was used to remove fish from their home tanks into a small portable tank. This tank was brought to a darkened behavior testing room, where fish were gently delivered into an observation tank (20 cm × 12 cm × 5 cm – L × H × W). During the observation time, a white light LED (i-bar LED lamp, Koncept) provided uniform illumination from above, such that the tank area was visible and the experimenter was obscured. The subjects’ behavior was recorded by an external Agent v5 HD web camera placed approximately 50 cm in front of the tank. The experimental protocol involved 8 minutes of acclimation, followed by 2 minutes of video recorded observation before CSE administration (before), and 8 minutes of video recorded observation that included 30 seconds of CSE delivery (after).

At the end of the experiment, subject fish were netted, and euthanized by immersion in ice water. Fish were dried using KimWipes, and frozen in dry ice immediately. Samples were kept at −80 °C until thawed for cortisol extraction.

### Behavior analysis and automatic quantification

For analysis of the behavior, recorded videos were first digitized at 20 frames/second. The frames were converted to 8-bit grayscale thresholded images by subtracting the background on ImageJ. Fish position was then tracked automatically on MetaMorph 6.3 using the “track objects” algorithm. Custom-written macros in Excel were used next to derive absolute position in the tank and speed in mm/sec information for each subject. Parameters used to identify alarmed state included velocity, time spent in the bottom quarter area of the tank, immobility episodes or freezing bouts, and darting episodes defined as erratic swimming episodes where the swim speed exceeded the normal swim speed by at least 8 standard deviations. As medaka slowly drift even when they stop swimming actively (Supplementary Video S1), an immobility episode was defined as <7 mm of displacement in 1 second. This was derived by taking 1/5th of the average swimming speed of 10 fish obsered for 10 minutes (~35 mm/sec) and was rare (less than 3 episodes on average) in a 2-minute observational time-bin before exposure to CSE.

### Statistics

Non-parametric statistical analyses were performed on R Studio or SOFA2 (http://www.sofastatistics.com/home.php). The Kruskal-Wallis H test was used to test for differences in multiple time bins and the pairwise Mann-Whitney U test was used when a difference was detected. Effect sizes were calculated using Cohen’s d.

### Consepecific Skin Extract (CSE) preparation

*Schreckstoff* was prepared as described for zebrafish with minor modification^42^. Briefly, fish were euthanized by immersion in ice-cold water. Shallow lesions (7–10) were made using a Sharpoint knife (22.5° stab). Fish were then immersed into 2 ml of fish water to 2 minutes and rocked on a rocker. The 2 ml crude extract was then centrifuged at 13.2 k rpm, filtered, and heated overnight at 95 °C. 250 ul of this extract was diluted in 750 ul of fish water and delivered into the testing tank gently at approximately 2 ml/min during the experiment such that no bubbles or disturbance were created.

### Cortisol extraction and measurements

Low quantity of extractable cortisol precluded comparison of changes in cortisol levels in the same subject during the course of the experiment and needed to be performed terminal endpoint. To minimize cortisol degradation, fish were netted after the behavior recordings, euthanized by immersion in ice-cold water, dried with KimWipes, and flash frozen in dry ice within 30 seconds. 2 control and experimental subjects could not be processed in this manner and were not used for further analysis. Therefore, N = 16 fish per condition. 8 fish were used for home tank condition.

Cortisol extraction was performed as described in^42^ with minor modifications. Briefly, for cortisol extraction, fish were thawed from −80 °C, and weighed, and the wet body weight was used for subsequent calculation of cortisol amount/gm. The fish body was dissected into five pieces and divided into five 2 ml Eppendorf tubes. 200 μl of 1X Phosphate Buffered Saline (PBS; pH 7.2) at 4 °C was added to each tube and their content homogenized using an Ultra-Turrax Disperser (T10 basic; IKA). Cortisol was extracted in 1400 μl of Ethyl Acetate as recommended by^56^ by vortexing the tubes for 30 s, followed by centrifugation at 7000 G-force for 15-min at room temperature. The organic layer that contained cortisol was collected in fresh tubes and left in the fume hood overnight to allow ethyl acetate to evaporate. Any remaining ethyl acetate was air dried gently. The precipitate was reconstituted in 1 ml of 1X PBS and stored at 4 °C. Enzyme Linked Immuno- Sorbent Assay (ELISA) was performed using Cayman cortisol measurement kit (Item # 500360) following the manufacturer’s instructions within 2–3 days of extraction and plates were read on a Tecan Infinite© at 420 nm.

### Histology

Three adults each of zebrafish and medaka were fixed in Bouin’s fixative solution (Sigma HT10132) overnight at room temperature. Whole-body 10 um transverse sections were prepared for all samples. Processing, wax sectioning, staining with Periodic acid Schiff’s reagent (PAS) and counterstaining with hematoxylin for all samples were performed by the Advanced Molecular Pathology Laboratory (AMPL), IMCB following standard protocols. Images of sections were collected on Zeiss AxioImager Z1 upright microscope.

## Additional Information

**How to cite this article**: Mathuru, A. S. Conspecific injury raises an alarm in medaka. *Sci. Rep.* **6**, 36615; doi: 10.1038/srep36615 (2016).

**Publisher’s note:** Springer Nature remains neutral with regard to jurisdictional claims in published maps and institutional affiliations.

## Supplementary Material

Supplementary Video 1

Supplementary Information
