# Supplementary material for: Conspecific injury raises an alarm in medaka
Source: Sci Rep. 2016 Nov 8;6:36615. doi: 10.1038/srep36615 (PMC5100478; doi:10.1038/srep36615)
Supplement: Supplementary Information [file srep36615-s2.pdf]

Title: Conspecific injury raises an alarm in medaka

Author: Ajay S Mathuru[1, 2 *]

Affiliation:
[1] Yale-NUS College, 12 College Avenue West, #01 - 201, Singapore – 138610
[2] Mechanisms Underlying Behavior, IMCB, 61 Biopolis Way, Singapore - 138673.

Correspondence:
Yale-NUS College,
12 College Avenue West, #01 - 201
Singapore – 138610

ajay.mathuru@yale-NUS.edu.sg ,
ajaym@imcb.a-star.edu.sg

Supplementary Information

Supplementary Figure 1

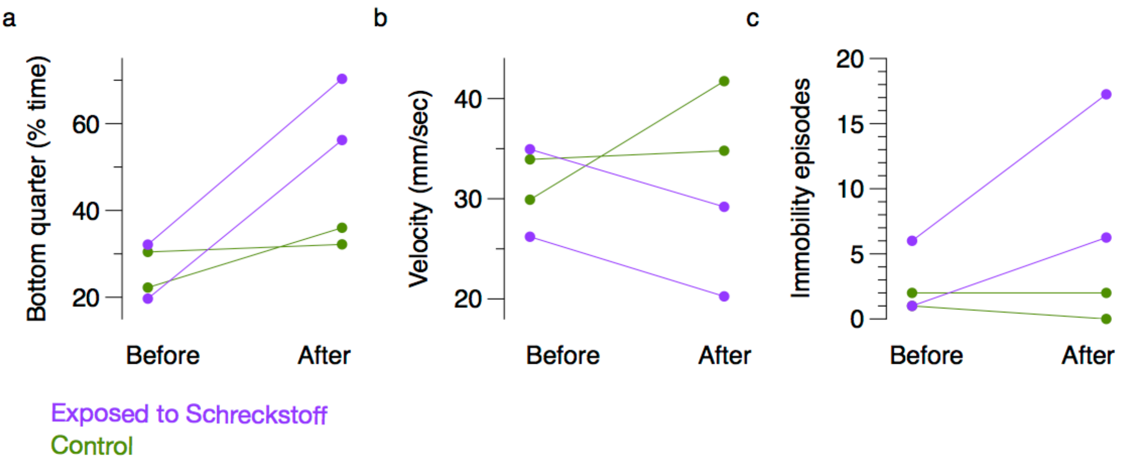

Behavioral measures comparing mean response before (left) and after (right) in controls (green) and those exposed to *Schreckstoff* (purple) for a) percentage of time spent in the bottom quarter, b) velocity in mm/sec and c) number of immobility episodes in a pilot

experiment. *Schreckstoff* exposed fish show an increase in time spent at the bottom of the tank, swim slower and show an increase in the number of episodes of immobility.

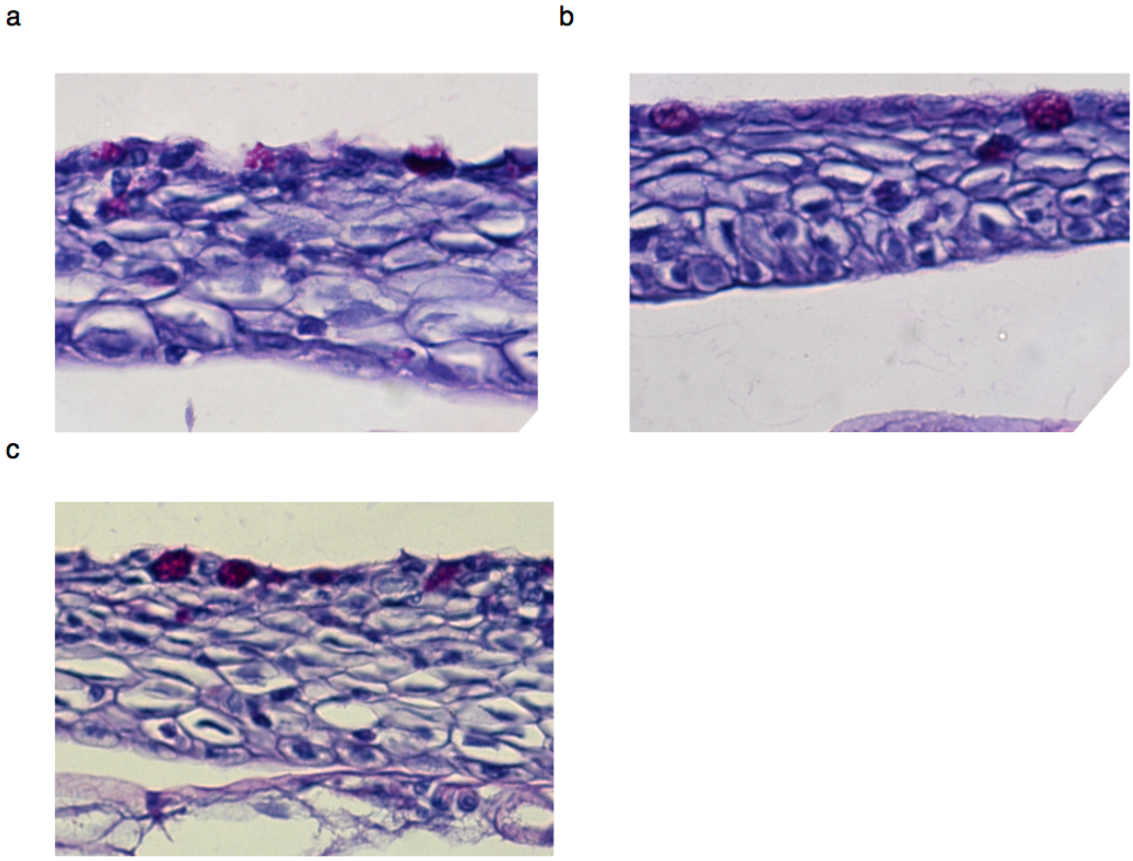

Supplementary Figure 2

Histology sections with PAS staining and haematoxylin counterstaining from 3 individuals.

Supplementary Video S1

Video shows a typical response of an individual subject before and after (indicated by a red LED in the left corner) exposure to the conspecific injury-released alarm substance (*Schreckstoff*).
